# Prenatal Exposure to the Pesticide DDT and Hypertension Diagnosed in Women before Age 50: A Longitudinal Birth Cohort Study

**DOI:** 10.1289/ehp.1205921

**Published:** 2013-03-12

**Authors:** Michele La Merrill, Piera M. Cirillo, Mary Beth Terry, Nickilou Y. Krigbaum, Julie D. Flom, Barbara A. Cohn

**Affiliations:** 1 Department of Environmental Toxicology, University of California, Davis, Davis, California, USA; 2 Department of Preventive Medicine, and; 3 The Metabolism Institute, Mount Sinai School of Medicine, New York, New York, USA; 4 Public Health Institute, Child Health and Development Studies, Berkeley, California, USA; 5 Department of Epidemiology and the Herbert Irving Comprehensive Cancer Center, Columbia University, New York, New York, USA

**Keywords:** blood pressure, DDT, hypertension, life course, prenatal

## Abstract

Background: Elevated levels of the pesticide DDT (dichlorodiphenyltrichloroethane) have been positively associated with blood pressure and hypertension in studies among adults. Accumulating epidemiologic and toxicologic evidence suggests that hypertension during adulthood may also be affected by earlier life and possibly the prenatal environment.

Objectives: We assessed whether prenatal exposure to the pesticide DDT increases risk of adult hypertension.

Methods: We examined concentrations of DDT (*p,p*´- and *o,p*´-) and its metabolite *p,p*´-DDE (dichlorodiphenyldichloroethylene) in prenatal serum samples from a subset of women (*n* = 527) who had participated in the prospective Child Health and Development Studies birth cohort in the San Francisco Bay area while they were pregnant between 1959 and 1967. We surveyed daughters 39–47 years of age by telephone interview from 2005 to 2008 to obtain information on self-reported physician-diagnosed hypertension and use of hypertensive medication. We used multivariable regression analysis of time to hypertension based on the Cox proportional hazards model to estimate relative rates for the association between prenatal DDT exposures and hypertension treated with medication in adulthood, with adjustment for potential confounding by maternal, early-life, and adult exposures.

Results: Prenatal *p,p*´-DDT exposure was associated with hypertension [adjusted hazard ratio (aHR) = 3.6; 95% CI: 1.8, 7.2 and aHR = 2.5; 95% CI: 1.2, 5.3 for middle and high tertiles of *p,p*´-DDT relative to the lowest tertile, respectively]. These associations between *p,p*´-DDT and hypertension were robust to adjustment for independent hypertension risk factors as well as sensitivity analyses.

Conclusions: These findings suggest that the association between DDT exposure and hypertension may have its origins early in development.

Hypertension is a costly and debilitating health problem in countries worldwide, and the prevalence of hypertension is rising. Population prevalence rates of > 20% have been reported in the United States, Venezuela, South Africa, and Lebanon, and rates of close to 50% have been reported in Germany and Spain (Arnaout et al. 2011; Hernandez-Hernandez et al. 2000). Increases in the prevalence of hypertension are generally attributed to changes in the prevalence of known preventable risk factors and to demographic shifts in populations. There is growing evidence that chemical exposures may also be associated with hypertension. For example, in a cohort study of pesticide applicators occupationally exposed to dichlorodiphenyltrichloroethane (DDT), mean serum levels of DDT and its metabolite dichlorodiphenyldichloroethylene (DDE) were significantly higher in persons who subsequently developed hypertension than in those who were normotensive (Morgan et al. 1980). More recently, the concentration of *p,p*´-DDT in breast milk was associated with increased maternal systolic blood pressure (Siddiqui et al. 2002). Despite the ubiquity of DDT and DDE in human tissues due to malaria prevention and environmental contamination, the possible contribution of these exposures to the global spread of hypertension has not been quantified.

There is extensive evidence that the environment during early development influences the risk of adult hypertension. In humans, low birth weight is associated with increased prevalence of adult hypertension (Barker et al. 1990; Leon et al. 1996). In animals, prenatal exposure to undernutrition, high-fat diet/adiposity, and glucocorticoids have all been associated with increased hypertension in offspring (Alwasel and Ashton 2009; Armitage et al. 2005; Gwathmey et al. 2011). These findings gave rise to the developmental origins of health and disease model, where “causes to be identified are linked to normal variations in fetoplacental and infant development” (Barker 2012).

Our *a priori* hypothesis was that prenatal exposure to DDT increases the risk of hypertension in adult life. We estimated the association between adult hypertension and prenatal DDT exposure in a prospective multiyear birth cohort study of women in the San Francisco Bay area, California, followed longitudinally from the prenatal period into their fifth decade (mean follow-up, 43.7 years).

## Methods

*Population*. The Child Health and Development Studies (CHDS) is a pregnancy cohort designed to examine the association between prenatal exposures and health and development over the life course for parents and children. The CHDS recruited women residing in the Oakland, California, area who were members of the Kaiser Permanente Foundation Health Plan and who sought obstetric care for pregnancies between 1959 and 1967 (van den Berg et al. 1988). CHDS participants gave oral informed consent for an in-person interview and collection of blood specimens at the first prenatal visit, in each trimester, and shortly after delivery (usually within 1–3 days), and gave permission for access to their own medical records and also their children’s medical records.

The present study of prenatal DDT exposure and adult hypertension used data collected for a cohort study of early-life predictors of breast density that was nested within the CHDS cohort. In 2005, two groups of adult daughters of CHDS participants were recruited for the breast density study: *a*) those who had at least one sister enrolled in the CHDS (to facilitate control of family-level confounders such as socioeconomic status), and *b*) those who had a birth weight greater than the 90th percentile (> 3,835 g) for live-born, singleton female births in the CHDS cohort (to facilitate an analysis of the association between high birth weight and breast density) (Terry et al. 2011). Additional eligibility requirements were available data on birth length and weight, at least one same-day childhood height and weight measure between 6 months and 5.5 years of age, maternal interview data, and prenatal serum. The breast density study was conducted with daughters’ informed consent at the time of their telephone interview and was approved by the institutional review boards of Columbia University and the Public Health Institute; the latter also oversees the continuing research based on the original families in the study and their now adult offspring. Mount Sinai School of Medicine deemed the present study not human subjects research. This study is in compliance with all federal guidelines governing studies of human participants.

*Variables*. At the CHDS baseline, each mother was interviewed in person to determine her race/ethnicity, parity, age at the time of pregnancy, date of last menstruation, prepregnancy body mass index (BMI), country of birth, and educational attainment. BMI at the first prenatal visit and gestational weight gain over the course of pregnancy were determined from maternal medical records. Maternal medical records were further examined to identify maternal physician-coded diabetes and preeclampsia, as well as daughters’ birth weight. We determined gestational age from mother’s report of last menstrual period and medical record report of infant’s date of birth. We evaluated daughters’ birth weight as a continuous variable and categorized as low (< 2,500 g) or normal according to the World Health Organization definition (Blanc and Wardlaw 2005).

We obtained data from adult daughters during telephone interviews conducted in 2005–2008 (Terry et al. 2011). Daughters reported their diabetes status, weight, height, race/ethnicity, educational attainment, annual household income, age, and menopausal status. Diabetes status was defined as a doctor ever or never telling a participant she had diabetes. Annual household income included salaries and all other sources. Participants were deemed premenopausal if they had a menstrual period within the preceding 12 months, perimenopausal if they had 60 days of amenorrhea of the preceding 12 months or between 2 and < 12 months passed since their last menstrual period, and postmenopausal if they either reported surgical menopause or had their last menstrual period > 365 days before interview. We calculated the BMI (kilograms per meter squared) of daughters from their height and weight. For the primary analysis, we classified hypertension based on self-report of physician-diagnosed hypertension plus use of antihypertensive medication in response to the question “How old were you when you were first diagnosed with high blood pressure or hypertension?” Age of hypertension diagnosis was a self-reported response to the question “How old were you when you were first diagnosed with high blood pressure or hypertension?”

*Laboratory assays*. DDT-related compounds (*p,p*´-DDT, *o,p*´-DDT, *p,p*´-DDE; collectively herein DDTs) were solid phase extracted from one maternal sample collected during either during the third trimester or within 1–3 days of delivery between 1959 and 1967 (Brock et al. 1996; Cohn et al. 2003; Gammon et al. 2002). There is a high degree of correlation between organochlorine chemicals levels in samples collected across prenatal and neonatal periods within a woman (Longnecker et al. 1999). Maternal sera triglycerides and total cholesterol were analyzed enzymatically as described in prior publications (Cohn et al. 2007, 2010).

*Statistical analyses.* We categorized DDTs into tertiles for analyses, with tertile boundaries of 6.97 and 11.9 µg/L for *p,p*´-DDT, 0.24 and 0.51 µg/L for *o,p*´-DDT, and 37 and 54 µg/L for *p,p*´-DDE. We conducted bivariate analyses to examine associations between *p,p*´-DDT exposure and possible confounders while accounting for the possible dependence between siblings in the analysis sample, using multinomial generalized linear models for categorical factors (PROC GENMOD, PROC FREQ, SAS version 9.2; all programs from SAS Institute Inc., Cary, NC) and generalized linear models for continuous factors (PROC MIXED). Life table curves for time to medicated hypertension onset by *p,p´*-DDT tertile were drawn using the unadjusted data (PROC LIFETEST).

We estimated associations [hazard ratios (HRs)] between tertiles of prenatal DDTs and daughters’ hypertension with multivariable regression analysis of time to self-reported hypertension diagnosis based on the Cox proportional hazards model using robust variance estimation to account for the correlated nature of siblings (PROC PHREG). Participants who did not report age at hypertension diagnosis were censored at their age at interview. Our model selection strategy was designed to address a single research question: Is there evidence of an association between prenatal exposure to DDTs and hypertension?

First we estimated unadjusted HR for medicated hypertension among women in the second and third tertiles of each DDT compound (*p,p*´-DDT, *o,p*´-DDT and *p,p*´-DDE) compared with the lowest tertile (model 1). Additionally, we constructed three multivariable models adjusted for maternal factors (model 2), adult risk factors (model 3), and the combination of these factors (model 4) (Lucas et al. 1999). For model 2 we considered potential confounding by maternal race/ethnicity, age, parity, country of birth, BMI, triglycerides, total cholesterol, preeclampsia, and gestational weight gain. The final model was adjusted for maternal race/ethnicity (African American vs. all other races, or Hispanic) only, using a 10% change in the point estimates for DDTs as the criterion for retaining potential confounders in the model (Maldonado and Greenland 1993). For model 3 we considered daughters’ race/ethnicity (African American vs. all other races, or Hispanic), age at interview, menopausal status (pre-, peri-, or postmenopausal), BMI (continuous), and diabetes status (ever or never) as potential confounders, and retained BMI, diabetes, menopausal status, and race/ethnicity using *p* < 0.05 for the association between the covariate and hypertension as the criterion for retention. Last, we examined a multivariable model 4, combining early-life confounding (model 2) and contemporary risk factors (model 3).

Because *p,p*´-DDT has been associated with birth weight and decreased birth weight is associated with increased risk of hypertension, we examined whether birth weight mediated our observed association between prenatal *p,p*´-DDT exposure and adult hypertension (Leon et al. 1996; Lopez-Espinosa et al. 2011). We considered birth weight a mediator of the *p,p*´-DDT exposure if it reduced the *p,p*´-DDT effect size. In addition, to assess whether BMI modified the association between hypertension and *p,p*´-DDT exposure, we examined multiplicative interaction between prenatal *p,p*´-DDT exposure and either maternal or daughter overweight and obesity (BMI > 25) (La Merrill and Birnbaum 2011).

**Figure 1:**
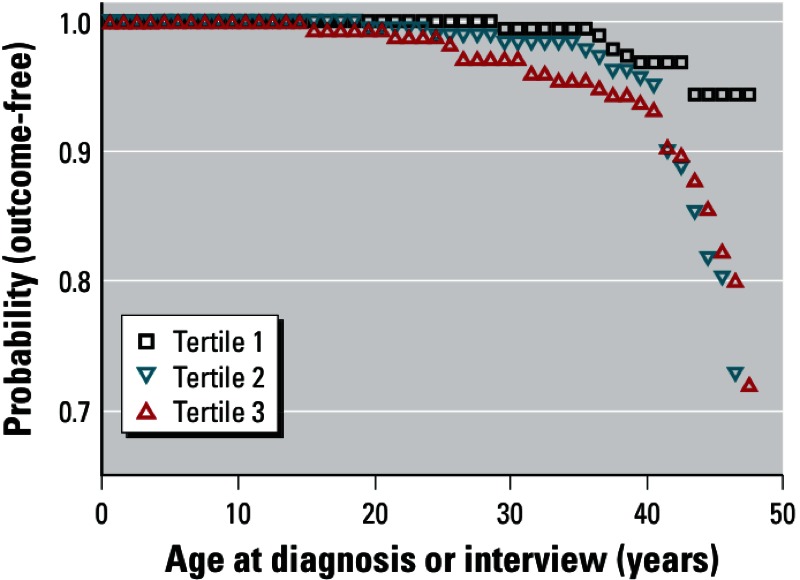
Unadjusted probability of being outcome-free as a function of prenatal *p,p´*-DDT tertile (*n* = 527 women). Outcome-free is defined by self- report of not having been treated for hypertension and censored at age at interview, whereas the outcome is defined by self-reported age at diagnosis of medicated hypertension.

We conducted sensitivity analyses of alternative DDT exposure variables dichotomized at the median or categorized into quartiles, and ran models that were mutually adjusted for all DDTs in addition to separate models of each. As evidenced from the unadjusted data in Figure 1, there was a nonlinear relationship between *p,p*´-DDT and medicated hypertension. We considered categorizing DDTs by the median, tertiles, and quartiles before multivariable analyses. Although all categories produced similar results, we grouped DDTs into tertiles to facilitate development of target exposure thresholds in public health practices while maintaining analytical power. We also estimated associations with all cases of self-reported hypertension, in addition to primary analyses of cases restricted to women who reported use of antihypertensive medications. Our *a priori* expectation was that the effect of *p,p*´-DDT exposure on hypertension risk would be strengthened in hypertension severe enough to warrant medication use because it is biologically plausible. To investigate whether oversampling high-birth-weight daughters influenced associations, we also modeled data after excluding the oversampled daughters with birth weight > 3,835 g. To investigate whether sibship influenced results, we also compared associations in the full sample with associations in all nonsisters plus *a*) the oldest sister from each sibling set or *b*) the second-born sister from each sibling set.

## Results

The CHDS recruited 15,528 mothers who received obstetric care through the Oakland, California, Kaiser Foundation Hospital for 20,754 pregnancies between 1959 and 1967. More than 90% of those eligible participated in the CHDS. From 2005 through 2008, 80% of the 1,526 daughters who were eligible for the breast density study were successfully traced, and 85% of traced daughters participated. Of the 527 women for whom DDTs, hypertension status, diabetes status, and BMI data were available, 156 were part of the high-birth-weight oversample, 70 were nonsisters, 301 were sisters (140 sets of pairs and 7 sets of “threes”), and 70 reported taking antihypertensive medication due to physician-diagnosed hypertension at the time of interview. Table 1 shows the distributions of potential confounders according to *p,p*´-DDT tertiles in the study population. Proportionally more African-American women were in the highest tertile of prenatal *p,p*´-DDT exposure than other women. Mothers’ age at birth and daughters’ age at interview were highest among women in the highest *p,p*´-DDT tertile.

**Table 1:** Distribution of maternal, early-life, and adult characteristics by prenatal *p,p´*-DDT exposure tertiles in 527 women of the CHDS [*n* (%) or LSM (95% CI)].

Characteristic	Tertile 1 (n = 168)	Tertile 2 (n = 182)	Tertile 3 (n = 177)
Maternal and early-life
Race/ethnicity
African American*	20 (26)	20 (26)	36 (47)
White, Asian, Hispanic, other	148 (33)	162 (36)	141 (31)
Maternal BMI (kg/m2)a	23.2 (22.7, 23.8)	23.3 (22.8, 23.9)	23.7 (23.2, 24.2)
Maternal cholesterol (mg/dL)b	245.0 (233.2, 256.7)	241.1 (229.6, 252.7)	264.0 (252.3, 275.8)
Maternal triglycerides (mg/dL)b	199.0 (187.7, 211.3)	201.7 (189.6, 213.8)	217.8 (205.6, 230.1)**
Maternal preeclampsia
Present	4 (36)	3 (27)	4 (36)
Absent	164 (32)	179 (35)	173 (33)
Maternal age at birth (years)	26.9 (26.0, 27.7)	26.6 (25.7, 27.4)	28.5 (27.6, 29.3)
Birth weight (g)			
≥ 2,500	166 (32)	175 (34)	173 (34)
< 2,500	7 (54)	4 (31)	2 (15)
≤ 3,835	123 (36)	115 (33)	107 (31)
> 3,835	50 (28)	64 (35)	68 (37)
Adult daughters
Race/ethnicity
African American*	21 (25)	22 (27)	40 (48)
White, Asian, Hispanic, other	152 (34)	157 (35)	135 (31)
Income ($US)c
< 25,000	11 (38)	6 (21)	12 (41)
25,000–49,999	31 (34)	31 (34)	28 (31)
50,000–99,999	62 (33)	56 (30)	70 (37)
≥ 100,000	57 (28)	83 (41)	62 (31)
Menopause
Premenopausal	129 (34)	133 (35)	119 (31)
Perimenopausal	27 (28)	31 (32)	38 (39)
Postmenopausal	12 (24)	18 (36)	20 (40)
Age at interview (years)	43.0 (42.7, 43.2)	43.8 (43.6, 44.0)**	44.3 (44.1, 44.6)**
BMI	27.1 (26.0, 28.2)	28.2 (27.2, 29.2)	27.7 (26.7, 28.8)
Diabetes status
Ever	9 (21)	18 (43)	15 (36)
Never	159 (33)	164 (34)	162 (33)
LSM, least square mean. an = 497. bn = 526. cn = 509. *p < 0.05 association of categorical variable across all p,p´-DDT tertiles (PROC GENMOD). **p < 0.05 LSM of linear variables in p,p´-DDT tertile 1 as reference group (PROC MIXED).			

The unadjusted risk of medicated hypertension was increased substantially among women with prenatal *p,p*´-DDT in the highest two tertiles of the study population (Figure 1). There were fewer cases of medicated hypertension among women in the lowest tertile of prenatal exposure to each of the DDTs compared with their respective higher tertiles (Table 2). The unadjusted prevalence of medicated hypertension which arose in women < 35 years old was 4.0% among women with prenatal *p,p*´-DDT in the highest two tertiles of the study population and only 0.6% in the lowest tertile. When we adjusted for confounding by maternal race/ethnicity in model 2, adjusted hazard ratios (aHR) for the middle and highest tertiles of prenatal *p,p*´-DDT exposure compared with the lowest tertile were 3.6 (95% CI: 1.8, 7.2) and 2.5 (95% CI: 1.2, 5.3), respectively. Consistent with expectations, medicated hypertension was associated with known risk factors including daughters’ race/ethnicity, menopausal status, BMI, diabetes, and age (data not shown), but only race/ethnicity, menopausal status, BMI, and diabetes were retained in the final model 3 based on *p*-values < 0.05, and associations based on this model were similar to those from model 2. Although both the model of early-life confounding (model 2) and the model of contemporary risk factors that predicted daughter’s hypertension (model 3) were both included in model 4 (mothers’ race/ethnicity, daughters’ race/ethnicity, menopausal status, BMI, and diabetes), only the covariables from model 3 remained significant, and the *p,p*´-DDT effect size remained comparable (Table 2). The other DDTs—*o,p*´-DDT and *p,p*´-DDE—were not significantly associated with medicated hypertension in any of the models (Table 2).

**Table 2:** Hazard ratios (HRs) of the association between prenatal DDTs and daughters’ medicated hypertension.

DDTs	Normotensive (n)	Hypertensive (n)	HR (95% CI) p-value			
			Model 1	Model 2	Model 3	Model 4
p,p´-DDTa
Tertile 1	160	8	Reference	Reference	Reference	Reference
Tertile 2	149	33	3.5 (1.7, 7.5) 0.0009	3.6 (1.9, 7.9) 0.0007	3.6 (1.8, 7.2) 0.0003	3.6 (1.8, 7.2) 0.0004
Tertile 3	148	29	2.9 (1.3, 6.4) 0.008	2.6 (1.2, 5.8) 0.02	2.5 (1.2, 5.3) 0.01	2.5 (1.2, 5.3) 0.01
o,p´-DDTb
Tertile 1	147	14	Reference	Reference	Reference	Reference
Tertile 2	157	28	1.7 (0.9, 3.2) 0.10	1.6 (0.8, 3.0) 0.17	1.3 (0.7, 2.5) 0.44	1.3 (0.7, 2.5) 0.43
Tertile 3	153	27	1.6 (0.8, 3.0) 0.15	1.3 (0.7, 2.6) 0.39	1.2 (0.6, 2.2) 0.57	1.2 (0.6, 2.2) 0.54
p,p´-DDEa
Tertile 1	149	16	Reference	Reference	Reference	Reference
Tertile 2	151	24	1.5 (0.8, 2.7) 0.24	1.3 (0.7, 2.5) 0.38	1.4 (0.8, 2.4) 0.30	1.4 (0.8, 2.4) 0.26
Tertile 3	157	30	1.7 (0.9, 3.2) 0.12	1.4 (0.7, 2.7) 0.30	1.6 (0.9, 2.9) 0.08	1.7 (1.0, 3.0) 0.06
Model 1: bivariate DDT analysis. Model 2 maternal covariables: race/ethnicity (African American/other). Model 3 daughter’s covariables: BMI (continuous), diabetes (ever/never), menopausal status (pre-/peri-/postmenopausal, race/ethnicity (African American/other). Model 4: model 2 + model 3. an = 527. bn = 52.						

Birth weight as a continuous measure was not significantly associated with medicated hypertension (model 3 aHR = 1.00; 95% CI: 0.99, 1.00), but low birth weight (< 2,500 g vs. ≥ 2,500 g) was (aHR = 3.3; 95% CI: 1.1, 9.9). However, associations between the second and third tertiles of *p,p*´-DDT and hypertension increased when adjusted for low birth weight (model 2 aHR = 4.0; 95% CI: 1.9, 8.2 and aHR = 2.9; 95% CI: 1.3, 6.2, respectively, and model 3 aHR = 4.0; 95% CI: 2.0, 8.0 and aHR = 2.8; 95% CI: 1.4, 5.9, respectively). There was also no significant interaction of either maternal (*p* = 0.6 for both tertiles 2 and 3) or daughter BMI (*p* = 0.8 and 0.9 for tertiles 2 and 3, respectively) on the association between prenatal *p,p*´-DDT exposure and medicated hypertension (model 3, data not shown).

We conducted secondary data analyses of alternative exposure classifications. Model 1 HRs for the association between prenatal *p,p*´-DDT and medicated hypertension were 1.9 (95% CI: 1.2, 3.1) for *p,p*´-DDT above the median of 9.00 ng *p,p*´-DDT/mL serum; compared with the lowest quartile of exposure, the HR for the second (6–9 ng *p,p*´-DDT/mL serum), third (9–13.39 ng *p,p*´-DDT/mL), and fourth quartiles (≥ 13.59 ng *p,p*´-DDT/mL) were 3.0 (95% CI: 1.1, 8.0), 4.5 (95% CI: 1.8, 11.6), and 3.2 (95% CI: 1.2, 8.7), respectively. Further, associations between *p,p*´-DDT and medicated hypertension based on a model mutually adjusted for all three DDTs (without other covariates) were similar to those based on model 1 (tertile 2 HR = 3.5; 95% CI: 1.7, 7.2 and tertile 3 HR = 2.6; 95% CI: 1.1, 6.1).

Associations with prenatal *p,p*´-DDT and hypertension were weaker when the case definition included all women with a self-reported diagnosis of hypertension (*n* = 111) (model 3 aHR = 2.1; 95% CI: 1.3, 3.5 and aHR = 1.5; 95% CI: 0.9, 2.7 for tertiles 2 and 3 relative to tertile 1, respectively) compared with associations with cases limited to women who reported using antihypertensive medication only (*n* = 70, Table 2). HRs increased when the oversampled daughters with birth weight > 3,835 g were excluded (model 3 aHR = 4.2; 95% CI: 1.9, 9.3 and 2.8; 95% CI: 1.2 to 6.9, respectively, *n* = 345). Last, the unadjusted association of prenatal *p,p*´-DDT with medicated hypertension was similar when the sample was restricted to nonsisters plus either the oldest (tertile 2 HR = 2.5; 95% CI: 1.1, 5.5 and tertile 3 HR = 2.4; 95% CI: 1.1, 5.3, *n* = 61 hypertensives) or second-born (tertile 2 HR = 2.6; 95% CI: 1.2, 5.9 and tertile 3 HR = 2.7; 95% CI: 1.2, 6.1, *n* = 52 hypertensives) sister per sibship (*n* = 380 total).

## Discussion

*Summary of principal findings.* We observed a positive association between prenatal *p,p*´-DDT and self-reported hypertension treated with medication in adult women that was robust to different modeling assumptions and adjustment for several known risk factors. Further, analyses of interactions between BMI and DDT were consistent with multiplicative joint effects.

*Comparison with other studies.* Although we know of no other studies that have examined the association between prenatal DDTs and offspring hypertension, blood pressure has been positively correlated with DDTs in the blood of pesticide workers in several studies (Morgan et al. 1980; Richardson et al. 1975; Sandifer et al. 1972). For instance, DDT was positively correlated with systolic blood pressure in a case–control study of South Carolina pesticide workers (Richardson et al. 1975). In another study of 120 occupationally exposed workers and 120 controls, systolic and diastolic pressures were positively correlated with DDT and DDE in plasma (Sandifer et al. 1972). In the largest occupational cohort study of the association between DDT and hypertension (*n* = 2,597), in which all participants were hypertension-free at baseline, the mean sum of serum DDTs was significantly higher in those who developed hypertension 4–6 years later compared with those who did not (Morgan et al. 1980). We know of only one previous study that assessed DDT congeners and blood pressure. Similar to associations observed here, the breast milk concentration of *p,p*´-DDT, but not of *o,p*´-DDT or of *p,p*´-DDE, was associated with increased maternal systolic blood pressure in Indian women who were admitted to a department of obstetrics and gynecology (Siddiqui et al. 2002), which together with our findings suggests that the structure of DDT congeners may influence their effects on blood pressure (Chaturvedi et al. 2010). Although our estimated association of hypertension with *p,p*´-DDT was consistently stronger for the second tertile than for the third, the lack of significance of this trend in our study is aligned with the linear trend in *p,p*´-DDT and systolic blood pressure that Siddiqui et al. observed.

*Potential biological mechanisms.* Experimental evidence suggests that DDTs can act on several arms of the renin angiotensin system (RAS) to possibly increase risk of hypertension. Gascon and Brodeur (1969) showed that angiotensin failed to contract guinea pig seminal vesicles *ex vivo* unless pre-treated with DDT. More recent studies have shown that Na-K-ATPase (sodium potassium adenosine triphosphatase) activity is reduced in prenatally programmed hypertensive rats (Alwasel and Ashton 2009; Armitage et al. 2005), and in parallel DDTs suppressed renal Na-K-ATPase in rainbow trout and bluegill fish (Campbell et al. 1974; Cutkomp et al. 1971; Davis et al. 1972). This body of experimental research together with our results suggest that future experimental studies should determine whether prenatal DDT exposure increases risk of hypertension in adult offspring.

*Strengths and weaknesses.* The CHDS is a prospective birth cohort with clear temporal separation between exposure and outcome. The CHDS pregnancies occurred near the peak of DDT use in the United States, allowing us to observe a large range of prenatal exposure. Further strengths of the study include clinical records of events during pregnancy, and quantitative assessment of prenatal DDTs from maternal sera.

The primary potential limitation of the study is the self-reported health status of the adult daughters. We conducted secondary data analyses to address this limitation. We expected that if prenatal *p,p*´-DDT exposure was a contributing cause of hypertension, prenatal *p,p*´-DDT effects would be more pronounced in more severe cases of hypertension. Indeed, we estimated stronger associations between prenatal *p,p*´-DDT exposure and self-reported hypertension treated with antihypertensive medication than associations with all cases of self-reported hypertension. Misclassification of covariables that were self-reported, such as daughters’ adult weight and height, used to calculate daughters’ BMI, could also be a potential limitation of this study. Although we were unable to internally validate self-reported BMI in the present study, comparisons of maternal and daughter adult weight, height, and BMI showed expected increases among daughters compared to the mothers’ generation for all three measures (data not shown), similar to secular trends observed nationwide (Nooyens et al. 2009; Reither et al. 2009). However, oversampling high-birth-weight daughters might have resulted in a selection bias that reduced the strength of the association between prenatal *p,p*´-DDT and adult hypertension.

Because DDTs were not measured during the life of the daughters and pharmacokinetic modeling has not been attempted, we cannot rule out the possibility that exposure after birth contributed to the association between prenatal *p,p*´-DDT exposure and adult hypertension. Although maternal exposure to DDTs during both gestation and lactation has been positively associated with fetal and neonatal DDTs in animals (Woolley and Talens 1971; You et al. 1999) and in human cord blood (Dorea et al. 2001), lactational *p,p*´-DDT exposure was uncommon for most CHDS daughters because of their 34% breastfeeding prevalence, which occurred for < 4 months duration in 60% of those who breastfed (Cohn et al. 2012). Thus our findings likely pertain to *in utero* exposure to *p,p*´-DDT in the majority of CHDS daughters, rather than lactational exposure.

*Public health implications.* DDTs remain an important environmental exposure. Each year, malaria affects 225 million people worldwide, and 781,000 infected people, mostly children, die (Agnandji et al. 2011). The World Health Organization recommends the use of DDT for indoor malarial vector control (Eskenazi et al. 2009). The mean serum 15.9 µg *p,p*´-DDT/L observed in children living in areas where DDT is used lies within the highest tertile of *p,p*´-DDT in this population (Herrera-Portugal et al. 2005). The present study suggests that people living in DDT-sprayed areas may have increased risk of hypertension due to their prenatal *p,p*´-DDT exposure. This adds to the existing literature suggesting that adult DDT exposure contributes to hypertension (Morgan et al. 1980). Because the distribution of malaria vectors may expand with climate change (Confalonieri et al. 2007; Rogers and Randolph 2000), climate change hazard assessment may benefit from inclusion of DDT toxicities, including further evaluation of DDT effects on hypertension.

Further, the persistence of DDTs coupled with climate dynamics suggests that people worldwide will continue to be exposed to DDTs that are already in the environment. DDT and its metabolite DDE have half-lives of about 4 and 10 years in people, respectively (Longnecker 2005). Due to their semivolatility, DDTs are found far beyond malaria-infested regions. For example, melting glaciers contributed 46% of the DDTs entering the Canadian Archipelago, and > 60% of the DDTs entering Canadian subalpine lakes (Blais et al. 2001; Macdonald et al. 2005). These environmental sources of DDTs bioaccumulate up the Arctic food chain such that intake of DDTs by Inuit people is comparable to the intake of DDTs by the general population in tropical regions with endemic malaria (Ritter et al. 2011).

## Conclusions

To our knowledge, our study is the first to suggest that hypertension in adult women may be increased as a consequence of early-life exposure to *p,p*´-DDT. Womb-to-adult studies of hypertension in humans are expensive and require long follow-up, thus making studies such as ours incredibly rare. Nevertheless, examining a larger study sample for an association between prenatal *p,p*´-DDT exposure and adult hypertension would be informative, and ongoing data collection in the CHDS cohort will provide this opportunity. Additional critical future research directions would focus on replicating our findings experimentally to evaluate potential hypertensive effects of *p,p*´-DDT exposure at different life stages, and on examining potential mechanisms of *p,p*´-DDT induced hypertension, including effects through the RAS.
